# Mycobacterial Arthritis at 19 Months After a Bacille Calmette–Guérin Vaccination

**DOI:** 10.4269/ajtmh.16-0618

**Published:** 2017-06-07

**Authors:** Chien-Yu Lin, Ye-Ming Lee, Jeng-Jung Chen

**Affiliations:** 1 Department of Pediatrics and Orthopaedics, Hsinchu Mackay Memorial Hospital, Hsinchu, Taiwan; 2 Division of Infection and Pathway Medicine, College of Medicine and Veterinary Medicine, The University of Edinburgh, Edinburgh, United Kingdom

**Figure 1.**
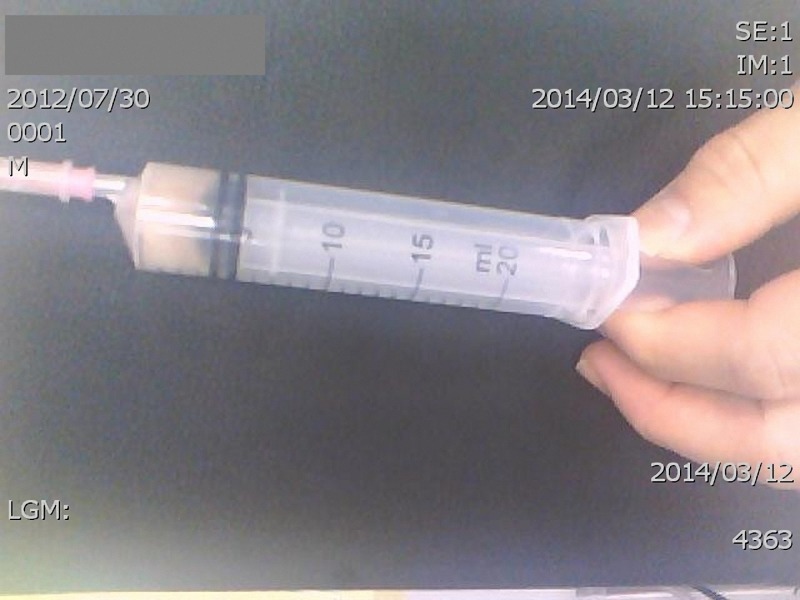
Arthrocentesis was performed, and characteristically cream-colored pus was drained. The pus culture yielded *Mycobacterium bovis*.

**Figure 2.**
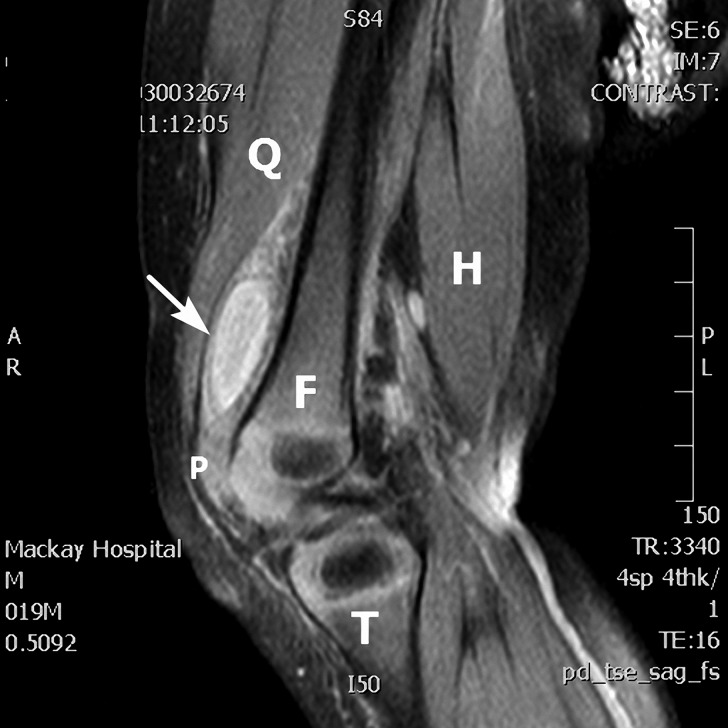
The sagittal view of T2-weighted knee magnetic resonance imaging shows abscess formation in the right suprapatellar bursa (arrow). The anatomic structures are labeled—F = femur, H = hamstring muscles, P = patella, Q = quadriceps muscles, T = tibia.

A 20-month-old afebrile boy living in northern Taiwan presented with swelling of the right knee for 5 days. No other respiratory or gastrointestinal symptoms were associated. A bacille Calmette–Guérin (BCG) vaccination had been performed when he was 1 month of age. The X-ray showed no bony destruction, and the sonography revealed some fluid accumulation in the right suprapatellar pouch. Arthrocentesis was performed, and characteristically cream-colored pus was drained (Figure 1

). The knee swelling persisted, and magnetic resonance imaging showed abscess formation in the right suprapatellar bursa (Figures 2

and 3

**Figure 3.**
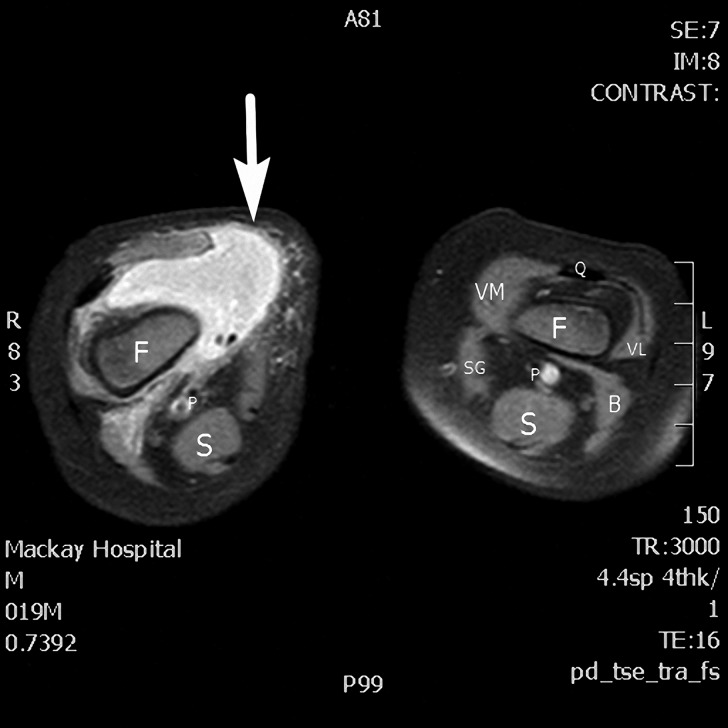
The axial view of T2-weighted knee magnetic resonance imaging shows abscess formation in the right suprapatellar bursa (arrow). The anatomic structures are labeled—B = biceps femoris, F = femur, S = semitendinosus and semimembranosus muscles, P = popliteal artery and vein, Q = quadriceps tendon, SG = sartorius and gracilis muscles, VL = vastus lateralis, VM = vastus medialis.

). Furthermore, surgical debridement was performed, and histological examination revealed granulomatous inflammation with caseation necrosis. The pus culture yielded *Mycobacterium bovis*. The patient was immunocompetent and was administered with isoniazid and rifampicin. He recovered without complications.

Tuberculosis remains an important health threat and the live attenuated BCG vaccine was used to prevent childhood tuberculosis. In 2009, the annual incidence of tuberculosis in Taiwan was approximately 69 cases/100,000 persons, and the Tokyo-172 BCG vaccination was included in the national immunization program with a coverage rate of 97%.^1^,^2^ BCG-related arthritis and osteitis is a rare but severe complication.^3^ The lower extremities are most commonly involved.^4^ The estimated incidence rates of BCG-related osteitis/osteomyelitis vary with place, time, and vaccine strain; for example, two cases per million vaccinations in Japan and 30 cases per million vaccinations in Finland.^4^,^5^ In Taiwan, the incidence was 12.9 cases per million vaccinations during 2005–2007.^2^ Pediatricians should remain alert for mycobacterial infections in children with BCG vaccination, including those administered long before presentation of arthritis, such as 19 months in the present patient. Early diagnosis and correct treatment are crucial for successful treatment.
